# “I smile, but Without Showing My Teeth”: The Lived Experience of Cleft, Lip, and Palate in Adults

**DOI:** 10.1177/1055665620922096

**Published:** 2020-04-30

**Authors:** Asgjerd Litleré Moi, Harald Gjengedal, Kari Lybak, Hallvard Vindenes

**Affiliations:** 1 Department of Plastic, Hand and Reconstructive Surgery, Haukeland University Hospital, Bergen, Norway; 2 Department of Health and Social Sciences, Western Norway University of Applied Sciences, Bergen, Norway; 3 Department of Clinical Dentistry, University of Bergen, Bergen, Norway

**Keywords:** lived experience, CLP, adults, phenomenology

## Abstract

**Objective:**

To explore and describe the experience of growing up with unilateral cleft lip and palate (CLP) in adults.

**Design:**

Face-to-face interviews. Giorgi’s phenomenological method was used for analysis.

**Participants:**

Twenty-one (mean age: 40.8 years) adults treated for unilateral CLP during childhood and adolescence participated in the study.

**Results:**

Growing up with CLP meant to become aware of bodily otherness. The possible reactions from peers early in life complicated the striving for inclusion outside the close family. Being self-confident, clever in school, physically fit, and having trusted friends represented barriers against teasing and bullying. Nevertheless, the reflected image, in mirrors, windows, and photos, reminded the participants of the objectifying looks from others and often led to bodily adjustments that persisted into adulthood. The trajectory of treatment was not questioned during childhood, and the participants accepted the decisions on care made by experts and parents. Although problems related to the cleft could persist or return after the termination of ordinary treatment, a more hesitant view on the possible benefits of additional surgery was typical in adulthood.

**Conclusions:**

In retrospect, growing up with a unilateral CLP was found to have been an unquestioned part of the adult participants’ childhood, a burden that they feared would, to some extent, also be passed to their own children. However, the CLP had not prevented them from achieving goals and satisfaction in life. The occurrence of persisting psychological, functional, and esthetic challenges in adults suggests the need for an individualized, lifelong, and multidisciplinary perspective on CLP follow-up.

## Introduction

Cleft lip and/or palate (CLP) is the most common birth defect, generating the need for multidisciplinary treatment and follow-up in approximately 1.5:1000 newborns worldwide (Sivertsen et al., 2008; Worley et al., 2018). A surgical treatment program starts in infancy, continues during childhood and adolescence, and frequently persists into adulthood. Oral clefts are related to reduced maxillary tooth status and difficulties with swallowing, articulation, hearing, growth, and appearance. Thus, children with CLP will experience the need for interdisciplinary support from plastic surgeons, orthodontists, dentists, otorhinolaryngologists, speech therapists, social workers, psychologists, and nurses. This team of health professionals works closely with parents and families aiming for the best possible oral- and esthetic health, functioning, and quality of life for those afflicted (Klassen et al., 2012).

Most studies of patients with CLP aged 21 years or less have reported the patients to have health-related quality of life and physical health comparable with normative control groups, and similar or even better levels of overall psychological and social health (see reviews Klassen et al., 2012; Aravena et al., 2015). On the other hand, patients with CLP may experience significant difficulties in communication competencies, self-concept, psychological distress, cognitive function, and social adjustment (Klassen et al., 2012; Aravena et al., 2015; Stock & Feragen, 2016). Moreover, in children aged 11 to 14 years, significantly reduced oral health-related quality of life has been reported (Kortelainen et al., 2016).

While knowledge about the child and adolescent experiences of life with CLP is steadily increasing, less is known about the patient-reported long-term outcomes. Some studies have described equal or even increased satisfaction with life in adults with CLP (Cochrane and Slade, 1999; Pisula et al., 2014). In contrast, a recent meta-analysis reported impaired health-related quality of life, mental health, and vitality in adults having an orofacial cleft (Queiroz Herkrath et al., 2015). Such challenges have been associated with social issues, including romantic relationships, education and employment, as well as the termination of treatment (Stock et al., 2015, 2016). Persistent and new challenges in adult patients with CLP include experiences of suboptimal results of surgery or unexpected psychological reactions, for example, bringing back unpleasant childhood memories of traumatic treatment procedures (Stock et al., 2015). Moreover, termination of treatment has been related to lack of information, knowledge on the entitlement to treatment and continuity during service transitions, as well as lack of competence at the local health services. Even though the interindividual variation seems to be substantial and psychological adjustment may fluctuate over time, support from psychotherapists and other health personnel may be of significant value for patients with CLP in adulthood and old age (Stock et al., 2016; Kappen et al., 2019).

The aim of this qualitative interview study was to explore and describe the patient-reported experiences of life of adults treated for unilateral CLP, including the experience of the impact of CLP and the treatment trajectory on their overall satisfaction with life.

## Material and Methods

### Study Design

In order to explore the lived experience of life with CLP, a phenomenological descriptive approach, inspired by Amadeo Giorgi (2009), was chosen. Phenomenology is characterized by being open to what is given, in this study, what is told by the participants. Thus, the researchers sought to bracket past knowledge, without questioning the existence of what was told (Giorgi, 2009). In this respect, the researchers must be especially aware of their own presuppositions and be open to meanings not corresponding to their theoretical and clinical experiences. The first author, who conducted most of the interviews and analysis in this study, was a critical care nurse with many years of experience of both research and clinical practice related to plastic surgery patients. However, she had no previous experience with patients with CLP, which may have helped her to adopt an open attitude during the research process. Collaboration with the CLP multidisciplinary team facilitated the questioning of data. The phenomenological understanding also offered a perspective overcoming body–mind and body–world dualisms, acknowledging human consciousness as the center of knowledge, in which the self and world of other people and things can only be fully understood in their interconnection (Zahavi, 2010). In addition to the phenomenological perspective, the application of methods and the presentation of findings followed the criteria of Standards for Reporting Qualitative Research (O’Brien et al., 2014).

### Participants and Setting

A cohort of 30 persons being treated for complete unilateral CLP from 1973 to 1979 was invited to participate in this study as part of a multidisciplinary follow-up including examinations by plastic surgeon, orthodontist, otorhinolaryngologist, and speech therapist. They all had their first surgical treatment at the age of 3 months and closure of the palate at the age of 2 years. The treatment protocol and the first data on treatment and follow-up of these patients were reported in 2009 (Tindlund et al., 2009).

In total, 21 of the patients consented to participate in the current interview study. Of the 9 patients who did not become participants in the study, we were unable to get in contact with 6, whereas 3 did not want to participate to this part of follow-up. All participants were Caucasian Norwegians, the mean age at the time of interview was 40.8 (range 37-44) years, 12 were men and 9 were women, 16 were married or cohabiting, and 18 had on average 2 (range 1-4) children. Most participants held an education equivalent to a bachelor’s degree (n = 10) or a master’s degree or higher (n = 5). The remaining 6 participants had less than 12 years of education. At the time of interview, 15 participants had paid employment, 3 received social security money, 2 were temporarily out of work, and 1 participant attended an occupational counselling program. About half of the group (n = 12) had no knowledge of relatives with an oral cleft in their family tree. The interviews were performed in a remote room or office at the hospital. All participants consented to have the interview digitally recorded. The interviews lasted on average 75 minutes.

### Data Collection

Two (A.M.L. and K.L.) of the authors performed all the interviews. The interview guide used in this study was based on a pilot interview with a patient with CLP from the same age group, followed by a discussion and a consensus in the local CLP multidisciplinary team. The participants were asked 3 main subjects, including (i) a description of their lives today, (ii) memories related to their CLP from childhood and adolescence, and (iii) the impact of their CLP on their lives today. Each main subject had from 9 to 14 subthemes assessing physical, emotional, social, and health-care aspects of the impact of CLP on their experience of life. The participants were asked to tell their stories as freely as possible, and the guide served mostly as a checklist to ensure that important themes were covered. An effort was made to keep questions and interruptions were to a minimum so that the chronology of the participants’ stories could facilitate recall. Probes and deepening questions were used to explore aspects perceived as valuable and important to their meaning of life. All interviews were recorded, transcribed verbatim, and prepared for analysis.

### Data Analysis

The analysis was conducted according to Giorgi’s descriptive phenomenological method (Giorgi, 2009). First, the source material consisting of 400 pages of transcribed interviews was read several times to get a sense of the whole. Next, the text was broken down by making highlights in the text where intuitive shifts in meaning occurred, identifying units of meaning describing the participants’ lived experience of CLP. Through this process, the researcher sought to maintain an open attitude, putting aside past knowledge, while searching for descriptions relevant to the research questions (Giorgi, 1997). Moreover, it meant not to question the existence of what was told. Meaning units were then described more directly from the perspective of health care. This is the heart of the method, carefully describing the features of the phenomenon, that is, what CLP had meant for the participants. The analysis was conducted for each interview separately, before identifying invariant meaning across all interviews and across the different contexts (Giorgi, 2009). Finally, the invariant meanings were presented in a general structure of meaning of life with CLP and given as interrelated themes. The coinvestigators were involved in the analytical steps to make sure that the meaning structure and themes were accessible and recognizable. NVivo 12 (QSR International, Southport, United Kingdom) was used to handle the voluminous data.

### Ethics and Approvals

The study was conducted according to the Helsinki Declaration (World Medical Association, 2013). All participants gave written consent after having received an invitation letter, informing them about the study purpose, contact information, voluntary participation, and the fact they could withdraw at any time before publication. The study was approved by the Regional Committee for Medical and Health Research Ethics (REC West; p.no. 2016/269).

### Findings

A general structure of meaning unfolded from the interviews of adults with CLP that included the following interrelated themes: to become aware of otherness, to struggle for inclusion, to integrate the looks of others, to adjust to things you have to do, to critically consider options for treatment, and to recognize own stories. Although with variation, they had learned to take life as it comes, not letting the CLP prevent them from reaching goals and life satisfaction in adult life. In the following, the themes are described in more detail:

#### To become aware of otherness

The first memories of having a cleft varied between the participants, both depending on age and circumstances. These memories were often about something that differed from their ordinary day-to-day life and were frequently connected to the participants’ needs for treatment. Some remembered traveling with family to the city where the hospital was located and considering it as almost a vacation.

And the flowers in the city, we always went there in May, when they were flowering, I believe it is my first memory, to travel by airplane and the flowers.

The first memories of the hospital could be associated with negative emotions, and in particular, with feelings of anxiety connected to being separated from their parents.

My first memory…I remember I lay in a bed having such metal rods, and I remember I was a little bit scared, I must have been about two years old. It was not common to have parents present all the time.

Moreover, hospitalization could be associated with a feeling of being different, like remembering not being included when the other children in hospital were playing.

The first time I thought of it, I was actually here (at hospital) […], I remember I looked down at myself, I had red clothes. And when you look down at yourself, you see your nose. So, I thought, this is why they don’t want to play with me.

What stood out as their first memory could also relate to other consequences of their condition, exemplified as the experience of milk coming out of the nose while drinking, or listening to their own way of speaking.

Yes, my first memory was sitting at my grandmother’s place listening to sound recordings of me, greetings we had recorded from our family to my grandmother, and when I listened to it, the lisping, my voice. I was so ashamed about my own voice and the sound of myself.

The recollection of their early speech generated great thankfulness to the speech therapist for valuable training and advice. Taken together, the content of the first memory of their cleft differed, but all memories included a sudden feeling of being pulled out of the ordinary day-to-day life and becoming aware of a kind of otherness.

#### To struggle for inclusion

At some point in their preschool or early school-age, the participants started to experience extra attention from peers, varying from minor curiosity to severe bullying. For children to be curious was generally regarded as unproblematic.

Kids are basically not bigoted, they are curious […], and I thought it was equally exciting to look at their palates, which were whole. I remember it as very odd; they were so smooth.

However, many had been afraid of bullies. Others remembered nicknames such as “flatnose” or “skewed-lip,” and peers imitating their speech, for example, repeating words that were difficult to articulate, for instance, words including the letter “S.” They could feel isolated from the rest of the class at school, and some endured in silence. The susceptibility to being teased or bullied was to some extent experienced as depending on their own self-confidence and ability to claim respect. Hence, fistfights were not uncommon during school age. One of the females described:

And then I started school, and it was another at school with a bilateral cleft, and we were teased a little bit. But I was quite strong, and I had a good upbringing together with the boys in the street, so I fought with the boys who teased me, even if they were older. They teased me about the cleft; it was the only thing that made me so angry.

Even later in life, some of the men described being physically fit as related to security.

The participants defending themselves by fighting were often met with a silent acceptance from adults, both in school and their parents. The role of the teacher in preventing bullying was crucial, but this was experienced very differently by the participants. Some teachers overlooked the child and never reacted to input from parents, whereas others collaborated with parents and gave invaluable support at school.

Besides the support from parents and teachers, being clever at school, a good footballer, having support from good classmates, having older and stronger friends, as well as having siblings or cousins at school were all mentioned as important ways of feeling protected against being bullied.

I perhaps got away with it easier than many others in my situation would have done, because I was not the typical person one bullies. I tried to ignore it, and I was not the most disadvantaged, or the most cautious person in the gang or in the class.

Even though the experiences of being excluded by peers varied, the recollection of childhood bullying could evoke strong feelings that persisted long into adulthood. Participants could have persisting problems with speaking loud or being understood in face-to-face conversations, conditions that could influence social interaction, as well as the choice of occupation in adult life.

#### To integrate the looks from others

Many participants mentioned appearance in terms such as “no big problem,” while at the same time saying that one had to accept one would never to be “the prettiest girl in class.” At the time they were newly born, their parents could have been recommended not to take pictures of their child and also to restrict visits from siblings. Some felt that their cleft was a taboo, and 1 participant had to ask her dentist for the only pictures she knew existed of her as a baby.

My dentist was very nice, and at my last consultation, he asked me: “Is there anything I can do for you, (name)?” Yes, I said, I would very much like to have the pictures you have of me before the cleft was closed.

Both during childhood and as adults, they could be hesitant about being photographed or they adjusted their appearance when photos were taken, like the experience of this man.

Of course, with regard to photos […]. One wishes to have a good smile, a winning smile. So, I smile, but like without (showing my) teeth.

Typically, the mirror also reminded them of the cleft.

I do check my profile in the mirror whenever I have the chance, and it is certainly the reason (the cleft).

Awareness of one’s own profile could also influence the preferred place of seating, for example, in a bus or plane, so they could show their best profile and hide the profile they appreciated least.

An example of what I would have experienced as very unpleasant is to sit next to someone on the bus. If they looked out of the window, they would see my face in profile; that is unpleasant.

All the participants appreciated and valued the results of surgery in regard to functioning and appearance.

Beside the operations when I was a baby, to move the jaw forward and avoid underbite and to correct the leaning nose I had before, to get it more straight, it has certainly done something for my self-image and self-confidence.

To a varying extent, the participants had noticed and interpreted stares from others both during childhood and as adults. People they met could still ask for an explanation of their appearance, for example, whether they had broken their nose or had been a boxer. Moreover, mirrors and pictures reminded them of the objectifying looks, causing some to adjust their habitual behavior, hide their smile, or change their profile.

#### To adjust to things you have to do

Surgical treatments and multidisciplinary follow-up by the cleft team were included in the participants’ childhood memories. Typically, their parents had prepared them briefly before each appointment, making little fuss about what was to come. They mostly had good memories of the hospitalizations and consultations.

It has been fun, exciting and safe.

They did not remember dreading the hospital consultations. Most importantly, parents usually stayed with them during the hospitalizations, making them feel safe.

I have no, or very few memories of hospital being negative. However, I did have dad with me, and he was extremely good at making things nice, and explaining when things were difficult. Yes, he was a very good person for support.

However, a feeling of sadness and insecurity among both parents and participants was described related to the situation when parents had been prohibited from being with their newborn during their first months of hospitalization. The mother of 1 participant, who was an only child, said that she did not know what it was like to have a baby, when she later became a grandmother. Another participant wondered whether it was the separation from her mother after birth or the separation from her nurse carers at the age of 3 months, which had caused her struggles with feelings of insecurity later in life.

I was here for three months (in hospital). And I think, for three months, the first three months of human life is important for bonding. So, I have thought a lot about that, what happened to the relationship with the family. Was this when I, in a way, lost my basic feelings of safety.

Another woman, who was separated from her parents for 2 months from postnatal day 3, stated that she believed this had perhaps made her more independent in life. However, as a mother she would never have managed to do the same after giving birth to her own children.

Overall, consultations with the cleft team were associated with being familiar with the same professionals over time and feeling recognized.

I think it was very okay (the consultations). I was talkative and spoke to all. I think they remembered me from one time to another, as much as I talked.

However, negative experiences also occurred, for example, feelings of anxiety when being held during painful procedures, bleeding, or not being prepared for the swollen and discolored face after surgery.

I understood it was my reflection in the mirror, but it was very scary as I looked awful. I looked like a sausage. And one of my hips was painful, so one hobbled on one foot. I had received no information,…hardly any information at all.

Listening to discussions about their condition and possibilities for treatment, and not being included in the dialog, could induce a feeling of not being seen, informed, and not knowing what to expect. Not all health professionals were equally experienced in communicating with and informing children. The consultations could then serve as a reminder of what was wrong with them, motivating them to quit treatment as soon as possible, wishing to get back to ordinary life where the cleft had less attention.

There were many people there in order to make you perfect. You get in there, and they weigh and measure and say exactly what is not perfect about you […] and they try this and that […] I mean, all these milestones are reminders of you being this way.

However, the treatments during childhood were something they had to undergo.

#### To critically consider options of treatment

While the participants remembered very few discussions about the treatment as children, they paid more attention as they became teenagers and young adults. The termination of treatment often took place at this age. Growing older meant to take more responsibility when discussing and deciding on treatment options, often with their parents and professionals, but sometimes against all advice. Several wished they had continued the follow-up by the orthodontist or dentist longer, as this might have prevented some of their dental problems as adults. So, they wished the professionals had been more persistent, convincing them to endure.

The advice to finish treatment should at least be given by the department of dentistry, by the clever ones. They should have taken the braces off, and told (name of the local dentist), that it is possible that it will slide, and what to do to stop it, and to make new appointments.

However, finishing treatment was also a relief.

To me, it felt very good, it was good to put an end to it, in a way. Now I am finished, and I feel finished too. […] For me, it was right to have this message; you make contact if you need us more. It was good to have the possibility, while at the same time as the program said; if I felt finished, I was finished. It is obvious it was kind of odd to walk out of the door here last time.

Problems related to the cleft could return after termination of the ordinary treatment schedule or persist into adulthood. The interviews revealed that at 40 years of age, many experienced difficulties such as recurrent sinusitis, ear infections, and impaired hearing, as well as obstructed nostrils causing a feeling of limited air supply. Difficulties with teeth, including biting and chewing also occurred. Others still had problems with articulation and increased mucus in their upper airways could add to this, making them avoid speaking in public. Like this businessperson said: “I have never enjoyed talking in big meetings because of my voice.”

The participants were used to living with impairments and evaluated them as ranging from minor to moderate. Thus, a renewed contact with the cleft team was often postponed. During the interview, the possibility of further corrections was mentioned and participants said they had weighed up the benefits and risks. “I have this fistula, which I can use to make a sound (makes a whistling sound) if I wish to, but it does not bother me.”

Pain and discomfort, as well as dissatisfaction with surgical results, could make them hesitant about asking for a new operation. Moreover, changed appearance following a new operation could also be an argument for declining further surgery.

It was a period when I considered it (an operation), because I knew I could have fixed it. Then I started to think, I do not want to when people know me as I am, I do not want to change appearance.

Moreover, becoming aware that the Norwegian social security system refunded costs for dental treatment related to the cleft and had led some to correct their teeth and have braces in adulthood.

While some of the participants would have appreciated being contacted by the cleft team on a regular basis, for example, every 5 years throughout life, in order to ensure the continuation of proper advice and treatment, others were satisfied with having the opportunity to make contact if needed. Some recommended that consultations with psychologists should be offered to teenagers on a regular basis. For most of the participants, the interview situation was the first time they had been asked for their subjective experiences of life with CLP.

Perhaps it might have been wise to have had this conversation earlier, on a regular basis […] Yeah, (to make contact by yourself) is more difficult. One will not be of any nuisance, one will not.

The participants mostly felt very satisfied with the results of the treatment they had been through, especially when comparing themselves to others who had experienced less fortunate results.

I am very satisfied with the job they have done. Certainly, I have been lucky the way things have turned out. So, I consider myself as fortunate when I see others having a lip-palate cleft, who have much more physical alterations, both speech and…

Information from the internet or patient organizations was used to a very small extent by the participants. However, they believed the internet could be of value to inform others in need of information about CLP.

#### To acknowledge own stories

The experiences of growing up with CLP was an integrated part of their life story and coming back to hospital to participate in the study gave rise to memories. Entering the hospital for the first time after the treatment period was finished could lead to a bodily awareness, recognizing that their childhood experiences were still part of them even today as adults.

Meeting the same professionals, entering the same offices, and having the same examinations were associated with positive feelings.

I enjoyed coming back. I remember I sat and read those things at the speech therapist when I was six years old, I think it is nice, it is a big part of me. But I cannot come back every 20 years. I have finished, and have ended it, and it is okay.

Typically, an urge to give information and reassure other families with a child with CLP, witnessed about feelings of knowledge and empathy developed over the years. Sitting next to small children born with clefts and their parents in the hospital waiting areas or reading about lack of treatment in developing countries could lead to feelings and recognition of own resources that could have been used to help others.

For parents of children born with a cleft, it would surely been reassuring to talk to someone like me, who has been through the whole trajectory, and in a way see that it all turns out well in the end

Based on their own experiences, they could also feel more empathy for children who were exposed to injustice in general. Moreover, most of them had no tolerance for those who bullies.

The participants were confronted with the impact of CLP on their lives when planning to have children themselves, and many had asked for signs of the cleft in their baby at the regular ultrasound checks. Irrespectively of whether they were a future father or mother, it seemed that they experienced more worries than their partners did. The time of birth could initiate thoughts on how to react and how to communicate with others if the newborn baby had CLP. Having a baby without a cleft was experienced as a relief since they knew that the baby would not have to go through the same ordeal as they had been through. Sometimes, the more children they had without a cleft, the riskier it felt to plan for a new pregnancy. Overall, they were prepared for what might come and would not let CLP in the child overshadow their gratitude for being a parent. Having the relevant knowledge and experience themselves, also meant they had more support to offer the child than other parents, who had never been confronted with a cleft before. Even so, feelings of guilt occurred when having a child with a cleft. “For me, I think it was very tough, I knew there was a risk that she could get it, and I felt a little guilty.” Hence, even though they had experienced significant challenges, many of which could persist into adult life, their lives were also stories of awareness, acceptance, and growth, acknowledging that their CLP had significantly influenced on their perspectives of life.

As adults, CLP was not associated with impairments precluding a good life, and whereas some thought about their cleft every day, many mostly forgot about it. If other illnesses or worries were present, this could influence their lives more than the cleft did.

It has never been a thing, and I have never needed it to be a thing. You would then make it into a problem, and it is not.

Overall, the CLP had not prevented the participants from achieving goals and being satisfied with life in adulthood.

## Discussion

During the interviews, the participants memorized discrete events of growing up with CLP, events that were integrated into their life stories. According to the Danish phenomenologist Dan Zahavi, self-awareness may be understood in 3 dimensions of self; an experiential dimension, an interpersonal dimension and a narrative dimension, and these dimensions are closely interwoven (Zahavi, 2010). Typically, the participants’ first awareness in childhood of a kind of otherness included memories from the hospital and recognizing cleft symptoms, such as difficulties with eating and drinking or disliking one’s own voice. Having to adapt to difficulties with eating seems to be a common concern in persons with CLP (Wong et al., 2013). Several studies also report on communication difficulties of patients with CLP in the pre- and primary school years (Klassen et al., 2012; Stock et al., 2018). The negative impact of problems of speaking and not being understood among peers seem to increase in early adulthood (Wong et al., 2013). The participants in our study remembered peers imitating their speech as very hurtful, and even at the time of the interview at 40 years of age, some participants struggled with speaking in larger groups of people.

According to phenomenology, self-awareness is embodied, given in a double sensation of interiority and exteriority as different manifestations of the same. Moreover, subjectivity cannot be understood without an essential, intrinsic reference to others through sharing the same world (Zahavi, 2005). The participants in our study felt the recognition of otherness as a barrier in their striving for inclusion among peers, especially during their childhood and adolescence. Peer teasing and harassment were related to appearance, and many participants remembered childhood nicknames based on the shape of their nose or lip. The unwanted awareness from others often increased during the school years and adolescence. As reported by others, teasing was mainly related to the appearance of the nose, lip, and teeth, as well as speech (Stock & Feragen, 2016; Wong Riff et al., 2018). The negative impact of teasing and bullying on the future psychosocial health of patients with CLP (Stock & Feragen, 2016) suggests that preventive measures in school may be important. In our study, the participants experienced that the classroom climate and support from teachers varied to a great extent; from teachers seeking knowledge and being supportive to teachers who never showed empathy or addressed teasing and bullying in the classroom. Stock et al have suggested that teachers need cleft-specific resources and training to address social, emotional, and communication barriers and must also understand the treatment and support transitions (Stock et al., 2018; Stock & Ridley, 2018). Our participants described personal and social factors that could limit or prevent teasing or bullying, experiences that are consistent with the reported association between lack of perceived teasing and the psychosocial resilience of patients with CLP (Feragen et al., 2009).

The looks that the participants perceived from others could influence behavior (Wong Riff et al., 2018) and could sometimes affect their habitual actions, for example, not showing their teeth when photographed or choosing a seat on public transportation allowing for their best facial profile to be visible to other people. These behaviors were only marginally conscious to the participants, and they seemed to become aware of them partly due to the forced self-reflection in the interview situation. Clarification of the role of conscious awareness of one’s own body has been sought through the use of terms such as “body image” and “body schema” as 2 distinct concepts (Gallagher, 1986). When consciously aware of the body, the participants typically referred to the body in parts, for example, the teeth, nose, and profile. The body image was then the perceived, understood, and emotional experience of the body. On the other hand, body schema has been described as the nonconscious performance of the body (Gallagher, 1986), as when the participants unconsciously positioned their bodies in public places or suppressed smiles showing teeth, revealing a partly unconscious bodily style adjusted to specific situations and environments. This in a way exemplifies how the 3 dimensions of self, others, and the world can only be fully understood in their interconnection, as has been stated in phenomenology (Zahavi, 2005).

The experience of the CLP formed the participants’ narrative of their treatments, mainly as adjusting to the things the participants just had to do. The memories of childhood hospitalizations reflected how the visiting policy for parents changed during the 70s and 80s; from a time when parent participation and open visiting hours for parents were uncommon to the later handing over of more care responsibilities to parents (Davies, 2010). As adults, the participants could still feel the loss associated with the early separation during the child’s first hospitalization. At a later stage, parents could feel anger from nurses for not being good enough caregivers in the hospital. A tendency among nurses to withdraw from interaction with the child when the mothers were present, has previously been described, which could add to parental feelings of lack of collaboration when the visiting policy became more open (Davies, 2010). However, the participants had mostly experienced close collaboration between their parents and the health professionals, allowing the parents to feel more secure and to rely on the team to normalize their child’s functioning and appearance (Nelson, Caress, et al., 2012; Nelson, Kirk, et al., 2012; Nelson & Kirk, 2013). The participants in the current study appreciated how their parents had strived to normalize their upbringing, focusing as little as possible on their CLP. Continuous support from parents had been crucial in the participants’ evaluations of achieving the best possible outcome.

Overall, the participants had a feeling of being recognized by the health professionals and they appreciated the familiarity they felt when consulting the same cleft team members over time. Overall, the participants felt very grateful for all the support they had received. However, feelings of being excluded from active participation during consultations had occurred and had induced an unpleasant feeling of being overlooked or of being different, perhaps reflecting a more hierarchical health-care system at the time (Davies, 2010; Alansari et al., 2014). The negative feelings of being overlooked or objectified should serve as an important reminder of the need always to talk to and informing the children and adolescents directly during the consultations.

To choose options of treatment was part of growing older and having a wish for autonomy. The participants felt relief when discharged from the standard CLP follow-up program. In accordance with other studies, dissatisfaction with own appearance impairing self-confidence seemed to be reduced during adulthood (Alansari et al., 2014). However, demand for treatment sometimes persisted, and new needs could arise long after the standard protocol was finished. The participants in our study therefore recommended that voluntary consultations with the cleft team should be offered when reaching adult age. Both the results of this study and experiences from the United Kingdom indicate that, even though clefts clinics take a life-span approach and allow for referrals after 20 years of age (Stock et al., 2015), adult patients with CLP weigh the benefits and risks of new surgery, often postponing new contact.

For many, this was the first time the participants had been asked to tell their story of having CLP. According to Arthur Frank, illness calls for stories in order to repair the damage that illness has done. “Stories are a way of redrawing maps and finding new destinations” (Frank, 2013). Illness stories are told as restitution stories, quest stories, or chaos stories, in which restitution stories have a recovery as focus, quest stories stress that something has been gained from the illness experience, for example, changes in internal standards and values, and chaos stories reflect that life never gets better (Frank, 2013). Overall, the most common story was a restitution story describing a movement toward health. In these stories, both the participants and their parents had been satisfied with being passive recipients of health care, confident in the outcome and making as little fuss as possible about treatment. Notably, in these stories, episodes of sufferings, for example, related to painful procedures, uncertainty, or anxiety were not displayed. In adulthood, these stories often included that the participants had achieved goals and were satisfied with life. Indications of quest stories, including growth gained from their experience of illness, were, for example, seen in participants’ memories from their childhood that made them more sensitive to injustice and bullying against children in general. Moreover, most of the participants had their own families and children at the time of the interview. Not all the participants knew of the risks of heritability. Even so, many had been worried during pregnancy and for those who had a child with CLP feelings of guilt occurred. However, the participants felt their own experiences were of help when the baby was born with a cleft (Stock & Rumsey, 2015). They also believed parents of newborns with a cleft could have benefited from hearing about their experiences and receiving their knowledge. Finally, elements of chaos stories also appeared. This could, for example, be related to wonderings about to what extent separation from parents early in life had inflicted on them in adult life. Some, experiencing limitations in daily life activities or reduced self-confidence could describe feelings of not being in control and not knowing how things could be better in the future. Hence, it is possible that health-care workers through active listening to CLP narratives and assisting patients in creating stories where they can feel in control may facilitate the improved outcome.

Understanding patients’ perspectives and narratives is crucial when it comes to developing optimal CLP treatment and follow-up. Whereas standardized questionnaires on patient-reported outcomes give an overview on group level of the participants’ functioning and well-being, unstructured interviews may give the participants an opportunity to express themselves in their own word (Polit & Beck, 2017). Thus, interviewing, although time-consuming, may give valuable supplemental and new information on the life experiences of patients with CLP. Consistent with such a view, the participants seemed to appreciate telling their stories, resulting in rich interview data. A possible limitation of this study may be that it did not include the whole spectrum of CLP. It is also possible that including more patients may have allowed for the identification of other significant experiences of living with CLP than those reported here.

The findings of this study indicate that continuity of care and meeting the same health professionals during the trajectory of CLP treatment were highly appreciated. The stories also indicated the value of including the patients in care and that the psychosocial aspects of having CLP perhaps were not fully assessed during adolescence. In Norway, there is no routine offering adult patients with CLP follow-up after the treatment is completed, making it up to the individual patient to contact the CLP team for reassessment. The persisting problems of many adults demonstrated in this study may suggest that this is a policy that should be reevaluated.

## Conclusions

Interviewing patients with CLP at the age of 40 years allowed for the unfolding of a general structure of meaning that included becoming aware of otherness, the struggle for inclusion, and the integration of the looks from others, as well as gradually gaining autonomy, which allowed them to make their own choices on treatment and follow-up. Although with variation, the participants had learned to take life as it comes, not letting the CLP prevent them from reaching goals and life satisfaction in adult life. The occurrence of persisting psychological, functional, and esthetic challenges in adults suggests the need for an individualized, lifelong, and multidisciplinary perspective on CLP follow-up.
